# Outcomes of a Structured Olfactory and Gustatory Rehabilitation Program in Children with Post-COVID-19 Smell and Taste Disturbances

**DOI:** 10.3390/jcm14010272

**Published:** 2025-01-06

**Authors:** Sami Khalid Almalki, Ahmed Mohamed Azzam, Saad A. Alhammad, Sami Alabdulwahab, Ahmed Ali Alshamrani, Abdulmajeed Nasser Alotaibi

**Affiliations:** 1 King Abdulaziz Specialist Hospital, Taif Health Cluster, Taif 26521, Saudi Arabia; abus7d33@gmail.com; 2 Department of Physiotherapy for Developmental Disturbance and Pediatric Surgery, Faculty of Physical Therapy, Cairo University, Giza 12613, Egypt; prf.ahmedazzam@gmail.com; 3 Department of Rehabilitation Health Sciences, College of Applied Medical Sciences, King Saud University, P.O. Box 10219, Riyadh 11433, Saudi Arabia; swahab@ksu.edu.sa; 4 Children’s Hospital, Taif Health Cluster, Taif 26514, Saudi Arabia; abdulmajeedna@moh.gov.sa

**Keywords:** olfaction, gustation, rehabilitation, exercise, COVID-19, occupational therapy

## Abstract

**Background/Objectives:** Severe acute respiratory syndrome coronavirus 2 (SARS-CoV-2) is closely related to SARS-CoV and uses angiotensin-converting enzyme 2 as its cellular receptor. In early 2020, reports emerged linking CoV disease 2019 (COVID-19) to olfactory and gustatory disturbances. These disturbances could be attributed to virus-induced damage to olfactory neurons or immune responses, thereby affecting sensory functions. This randomized controlled trial aimed to evaluate the effectiveness of a structured orofacial rehabilitation program in improving smell (olfaction) and taste (gustation) sensations in children post-COVID-19. **Methods:** Forty children recovering from COVID-19 in government hospitals in Saudi Arabia were included and randomly assigned to the control group or the experimental group. The orofacial program included (a) facilitation of olfactory function using the 40-item modified Arabic version of the University of Pennsylvania Smell Identification Test (UPSIT); (b) assessment of gustatory function using taste strips with four varying concentrations; and (c) orofacial myofunctional therapy. The intervention was applied three times a week and lasted for 3 months. **Results:** The experimental group showed a significantly greater improvement in UPSIT scores (median change of 24.1%) than the control group (14.7%; *p* = 0.010). However, no significant difference was found in the taste strip test scores among the groups or between male and female participants. **Conclusions:** This study suggests that a structured orofacial rehabilitation program could enhance olfactory and gustatory functions in children recovering from COVID-19.

## 1. Introduction

Severe acute respiratory syndrome coronavirus 2 (SARS-CoV-2) shares 80% of its genome with SARS-CoV and uses angiotensin-converting enzyme 2 (ACE2) as its cellular receptor [1]. The disease caused by SARS-CoV-2 is called “coronavirus disease 2019” (COVID-19) [2]. CoV contains four major structural proteins: envelope, membrane, nucleocapsid, and spike (giving it a crown-like appearance, and hence the name “coronavirus”). Compared to the binding capacity of the SARS-CoV receptor-binding domain (RBD), the SARS-CoV-2 RBD has a 10- to 20-fold greater binding affinity for ACE2. In addition, the RBD of SARS-CoV-2 binds to soluble ACE2 more strongly than the RBD of SARS-CoV. For this reason, COVID-19 spreads faster than SARS-CoV, which resulted in a continued increase in the number of cases [3,4,5]. Patients with COVID-19 reported symptoms such as fever, dry cough, tiredness, and shortness of breath, which were moderate in approximately 80% of cases. However, if the condition deteriorates, the patient might descend into respiratory distress or respiratory failure, necessitating treatment in an intensive care unit [6].

In February and March 2020, increasing frequencies of olfactory and gustatory disturbances in COVID-19 patients were reported for the first time [7]. Initially, these findings were informal; however, over time, the literature began to frequently report an increase in the incidence of chemosensory impairment in COVID-19 patients [8]. Loss of smell associated with COVID-19 may be due to virus-induced damage to olfactory receptor neurons on the olfactory epithelium or cytokine storm (immune cell disturbance and cytokine release), which affects the nervous system, including the sensory organs responsible for smell. Both ACE2 and transmembrane protease serine-2 protein receptors are necessary for effective SARS-CoV-2 infection. Chemosensory deficits have been identified as a particular COVID-19 indication [7,9].

The capacity to detect tastes in individuals with COVID-19 is significantly influenced by olfactory abnormalities, as both chemosensory systems interact closely. One hypothesis for acquired ageusia is linked to the impact of COVID-19 on ACE2 receptors in the taste buds. Many patients experience these effects, which can persist long after respiratory symptoms have subsided, posing a considerable medical issue [10,11]. A systematic review and meta-analysis of 38,198 patients across 104 studies found that the random prevalence estimates for olfactory dysfunction were 43.0%, for taste dysfunction were 44.6%, and for total chemosensory dysfunction were 47.4% [7].

Recent studies suggest that loss of smell and taste should be recognized as pediatric symptoms of COVID-19 and should always be tested, as they can be the sole manifestation of the infection [12,13]. In children with COVID-19, olfactory function tends to decline with age but improves over time after recovery. However, in some cases, olfactory sequelae may persist even longer after the illness has resolved [12]. In this study, we aimed to evaluate the effectiveness of a specific orofacial rehabilitation program in improving smell (olfaction) and taste (gustation) sensations in children post-COVID-19.

## 2. Materials and Methods

This randomized control trial was conducted to examine the efficacy of a specific orofacial rehabilitation program in improving the chemoreceptors of smell and taste sensations in children post-COVID-19, comparing the pre- and post-intervention study group with the control group.

### 2.1. Setting and Participation

The study included a total of 40 children recovering from COVID-19 in government hospitals in Saudi Arabia. The participants were divided into two groups: the control group (who received a specific orofacial rehabilitation program) and the experimental group (who received no rehabilitation program). The intervention was applied three times a week and lasted for 3 months.

### 2.2. Sample Size Estimation

Using G*Power version 3.1.9.4, we created a sample size estimated at an alpha level of 5%, power of 80%, and effect size of 0.25 in two different groups and two measures to assess differences in loss of smell and taste post-COVID-19 between children who received a specific orofacial rehabilitation program and those who did not receive the program. The required sample size was 40 participants, with 20 in each group and an exhaustion rate of 15%. The study used the probability sampling technique, specifically systematic sampling, which involved searching the Ministry of Health (MOH) database to identify post-COVID-19 children who met the inclusion criteria. Then, each child was assigned a number starting from 1, and every tenth number was selected to reach a total of 40 children. Subsequently, all children with odd numbers were placed into the control group, while those with even numbers were assigned to the experimental group.

### 2.3. Inclusion Criteria

The following children were included:

- Nonhospitalized post-COVID-19 Saudi children
- Between 12 and 18 years of age (male/female)
- Complaints of loss of smell and taste
- Completed the government-mandated 10-day quarantine
- Officially registered in the MOH database

### 2.4. Exclusion Criteria

The following children were excluded:

- Children who had not completed a specific orofacial rehabilitation program for 3 months
- <12 years of age
- Mental or neurological condition
- Cancer
- Allergy to University of Pennsylvania Smell Identification Test (UPSIT) or taste strips test (TST)
- Post-COVID-19 children with no complaints of smell or taste disturbances

### 2.5. Evaluation Procedure

At the physical therapy department, the participants’ demographic and anthropometric information was collected. A modified Arabic version of the UPSIT was then administered to all participants. A standardized microencapsulated odorant identification test was employed, in which 40 odorants were given in a “scratch-and-sniff” manner, with four levels for each odor. This test identifies normosmia when the child achieves 34 correct answers from a total of 40 items of UPSIT, mild microsmia for 30–33, moderate microsmia for 26–29, severe microsmia for 19–25, and anosmia for 6–18. Based on cultural adaptation and the suitability of use with children, benzene, smoke, motor oil, and natural gas were replaced with oud, ginger, cinnamon, and musk, respectively. TST was used to examine taste sensation; the strips featured four different tastes—sweet strips, salty strips, bitter strips, and acidic strips—with four different concentrations for each. Children in the experimental group were asked to taste the strips and then graded (1, 2, 3, or 4) according to the concentration level, where 4 indicates tasting the lowest concentration, 3 the second lowest concentration, 2 the second highest concentration, and 1 the highest concentration.

### 2.6. Treatment Protocol

The rehabilitation program was applied over 12 weeks, 3 days per week, and for a total of 30 min per session.

#### 2.6.1. Facilitation of Olfaction Function

This was done using the 40-item UPSIT (modified Arabic version).

#### 2.6.2. Facilitation of Gustatory Function

This was done using TST; four types with four progressive concentrations: sweet strips (concentrations of 0.4, 0.2, 0.1, and 0.05 g/mL of sucrose); salty strips (concentrations of 0.25, 0.1, 0.04, and 0.016 g/mL of sodium chloride), bitter strips (concentrations of 0.006, 0.0024, 0.0009, and 0.0004 g/mL of quinine hydrochloride), and acidic strips (concentrations of 0.3, 0.165, 0.09, and 0.05 g/mL of citric acid). We started with the same concentrations that the child could not recognize during the taste evaluation.

#### 2.6.3. Orofacial Myofunctional Therapy

The following were included:

1. Lip exercises [14]: Lip exercises are important for moving food around the mouth and producing a food bolus for ease of swallowing. Lips aid in creating a tight seal around the mouth, preventing food and liquids from spilling out. These exercises are performed as follows:
  - Inflate the cheeks with air and hold it in the mouth for 10–20 s. Repeat 10–20 times. This helps the lips to develop the ability to sustain a tight seal.
  - Using the hand, insert a flat, soft item between the lips and hold it without letting it fall.
  - For 10 s, keep the lips puckered (kissing). Repeat the exercise 5–10 times.
  - Maintain a smile on the face for at least 10 s.
  - Tongue exercises:
  - Tongue pushup
    Place the tip of the tongue against the hard palate on the upper jaw of the mouth, approximately behind the top teeth, then push upward and hold it for 5 s. Repeat 10 times.
  - Touch nose
    Stick the tongue out and touch the tip of the nose. Hold for 10 s, then relax. Repeat 10 times.
  - Touch chin
    Stick the tongue out and lick the lower half of the chin. Hold for 10 s, then relax. Repeat 10 times.
  - Push tongue left
    Stick the tongue out and move it as far as possible to the left. Hold for 10 s, then relax. Repeat 10 times.
  - Push tongue right
    Stick the tongue out and move it as far as possible to the right. Hold for 10 s, then relax. Repeat 10 times.
  - Tongue roll
    Roll the tongue by folding the edges toward the middle. Stick the tongue out as far as possible while keeping it folded. Hold for 10 s, then relax. Repeat 10 times.
  - Tongue pushup against a spoon
    Push the tip of the tongue firmly against a spoon held in front of the lips for 10 s while keeping the tongue straight. Repeat 10 times.
2. Nasalis muscle exercises (alar part and transverse part)
  - Nose wrinkle
    Wrinkle the nose and cheeks. Hold, then relax. Repeat 10 times.
  - Nose shift
    Shift the nose to one side and hold. Repeat on the other side.
  - Place two index fingers beside the nostrils and press them applying moderate pressure. Then flare and release the nostrils at a rate of one flare per second, against the resistance of the fingers. Repeat 30 times.

### 2.7. Statistical Analysis

One-sample Kolmogorov–Smirnov test showed that the UPSIT and TST scores were normally distributed (*p* > 0.05). Parametric statistical tests were, therefore, applied for comparisons.

## 3. Results

### Baseline Characteristics of the Participants

Table 1 summarizes the participants’ baseline characteristics, including age, gender, and body mass index.

Olfaction significantly improved 3 months post-COVID-19 in both groups (*p* < 0.001) (Table 2 and Table 3).

Gustation also improved significantly 3 months post-COVID-19 in both groups (*p* < 0.001) (Table 4 and Table 5).

As shown in Table 6, the percent change in the UPSIT scores post-COVID-19 was significantly higher in the experimental group than in the control group (median values of 24.1% and 14.7%, respectively; *p* = 0.010). On the other hand, there was no statistically significant difference between the two groups regarding the percent change in the TST scores 3 months post-COVID-19.

## 4. Discussion

Reports of olfactory and gustatory abnormalities in COVID-19 patients first emerged in February and March of 2020. While these observations were initially unofficial, over time, studies began to consistently report increased incidence of smell and taste impairment in these patients [6,12]. This affects the majority of patients, and the effects can continue long after the respiratory symptoms have resolved, representing a significant medical problem [8]. A wide range of behavioral and physiological responses associated with the acquisition, identification, and absorption of nutrition is caused by gustation and olfaction [15]. These senses provide vital details about environmental threats, such as fires and spoilt food, because they can decode chemical stimuli into neuronal stimuli and then determine a response accordingly [16].

In this study, we aimed to determine whether administering a specific orofacial rehabilitation program to post-COVID-19 participants would bring any significant value to accelerating their recovery from COVID-19-induced smell and taste impairments. Our findings demonstrated improved smell sensation in both groups, with the means and standard deviations (SDs) pre-test and post-test of 26.45 ± 4.85 and 30.50 ± 3.75, respectively, and pre-intervention and post-intervention of 26.20 ± 4.53 and 33.40 ± 3.10, respectively (*p* < 0.001). The percentage change indicated a significant difference in the experimental group compared to the control group. The median values for the experimental and control groups were 24.1% and 14.7%, respectively (*p* = 0.010). This suggested that the intervention (specific orofacial program) can improve olfaction status.

Taste function improved in both the control and experimental groups; however, there was no significant difference between them in terms of percentage change considering the means and SDs for the pre-test, post-test, pre-intervention, and post-intervention scores (6.35 ± 1.73, 9.95 ± 2.31, 7.80 ± 2.04, and 12.0 ± 2.60, respectively; *p* < 0.001). Furthermore, in view of gender-related effects on smell and taste impairments, the present study showed that, in terms of percentage change, there was no statistically significant difference between males and females. The smell sensation median values for males and females were 21.4 and 17.0, respectively (*p* = 0.581). Similarly, there was no statistically significant difference in taste sensation; the median values in males and females were 55 and 58.6, respectively (*p* = 0.459). This study showed that there was a negative correlation between the participants’ age and their present status of taste sensation improvement post-intervention in the experimental group (r = -0.352, *p* = 0.026). This suggested that a greater improvement in taste function is more likely in younger patients than in older patients. Hence, future research investigating the extent of our intervention’s effectiveness on adults and elderly patients in multilabel regions is warranted.

We used UPSIT in this study because it is one of the most widely accepted tests globally for assessing the sense of smell and has been validated in a variety of populations [17,18]. UPSIT has normative data for the combined age groups of 5–9 years and 10–14 years, with an acceptable test-retest reliability coefficient score of ≥0.7. Taste strips are arguably the best tool to examine taste function, with proven good reliability, validity, and practical technique when applied to a Portuguese population. TST has been used in numerous research and therapeutic situations and has also been shown to have strong test-retest reliability, as well as a high level of acceptability by children and adolescents [18,19,20].

UPSIT was used to show that decreased smell function is a significant indicator of SARS-CoV-2 infection. Some post-COVID-19 participants in the same study stated that the appearance of their other COVID-19 symptoms corresponded with or followed the onset of their olfactory impairment [2,17,21]. This raises the possibility that smell testing could, in some cases, be used to identify COVID-19 patients who need immediate medical attention or isolation. Despite the generally known fact that viruses and other xenobiotics can harm the olfactory neuroepithelium, the mechanism underlying SARS-CoV-2-induced loss of smell remains unclear. Persistent olfactory dysfunction is most often caused by acute viral upper respiratory infections that harm the epithelium, and many viruses are recognized to enter the brain via cellular and pericellular transport through this route. However, if the stem cell layer of the olfactory neuroepithelium is not severely injured, the olfactory neuroepithelium has a high capacity to regenerate and improve in function spontaneously over time [21].

Some studies have reported that the number of post-COVID-19 patients with complaints of loss of taste is lower compared to the number of those with complaints of smell dysfunction, which could be likely due to more damage to the olfactory epithelium than to the taste buds [10,11,14,22,23,24,25,26,27]. The various nerves that facilitate taste function seem to act in a compensatory manner among each other in case of injury or infection, which probably explains why taste loss is lesser in frequency compared to smell dysfunction [28,29]. Therefore, it can be hypothesized that the pathophysiology of taste abnormalities in post-COVID-19 patients could be attributed to indirect damage to taste receptors caused by infection of epithelial cells and subsequent local inflammation influenced by direct infection of the olfactory epithelium. This means there was faster recovery in gustation than in olfaction in the participants in the present study.

Evidence shows that early orofacial exercises helped minimize pain and tenderness [30,31], release trigger points, lengthen shortened muscles, and loosen the jaw’s limited movements in hospitalized COVID-19 patients with continuous positive airway pressure-induced orofacial pain and temporomandibular dysfunction [32,33,34].

Ponce-Campos et al. [34] conducted a physiotherapy program, comprising 12 sessions over 4 weeks, for 42 post-COVID-19 patients. They observed a percentage increase in the forced expiratory volume in 1 s (FEV_1_), an increase in forced vital capacity (FVC), and a decrease in the FEV_1_/FVC ratio. The basal saturation of oxygen-enhanced capacity and the maximal functional capacity also improved. They further observed that 6 min walk test performance increased by 13%, and handgrip strength showed an increase in both the left and right hands.

Given the significant improvement in olfactory function, as evidenced by the UPSIT scores, these programs can be considered a valuable therapeutic approach to address sensory impairments that may arise post-infection. Enhanced olfactory capabilities are crucial, not only for the enjoyment of food but also for overall quality of life, as they play a vital role in safety and environmental awareness. Thus, integrating such rehabilitation strategies into pediatric care could facilitate a smoother recovery process and improve daily functioning for affected children.

Moreover, the lack of significant differences in gustatory function highlights an important area for future research and program development. Understanding the reasons and the mechanisms behind this could lead to tailored interventions aimed specifically at enhancing taste sensations. By expanding the scope of rehabilitation programs to include targeted gustatory training, physical therapists and occupational therapists can better support children in their recovery journey. Ultimately, this study advocates for a holistic approach to post-COVID-19 rehabilitation, emphasizing the importance of sensory integration in restoring the well-being of young patients.

The limited number of patients included in the study was mainly due to the challenges of enrolling children and conducting all the clinical tests required by our protocol. Additionally, the lack of communication skills at this early age, along with difficulties in obtaining parental consent, logistics, and scheduling, further limited enrollment. We hope to share data on larger populations in the future. A second limitation is that only one region was targeted for data collection. Future research should focus on evaluating the optimal timing of the orofacial exercise program to determine its most effective implementation to restore the smell and taste. It is critical to consider how varying levels of exercise intensity and duration impact olfactory and gustation functions.

## Figures and Tables

**Table 1:** Baseline characteristics of the participants.

	Control Group N = 20	Experimental Group N = 20
Age (years)		
Mean	14.80	14.35
SD	1.94	1.90
Gender (frequency, %)		
Male	10; 50%	12; 60%
Female	10; 50%	8; 40%
BMI (kg/m^2^)		
Mean	20.89	21.93
SD	3.61	2.64

SD, standard deviation; BMI, body mass index.

**Table 2:** Average test of smell sensation in the control group.

Smell	Mean	SD	*p*-Value
Pre-test	26.45	4.85	<0.001
Post-test	30.50	3.72	

SD, standard deviation.

**Table 3:** Average test of smell sensation in the experimental group.

Smell	Mean	SD	*p*-Value
Pre-intervention	26.20	4.53	<0.001
Post-intervention	33.40	3.10	

SD, standard deviation.

**Table 4:** Average test of taste sensation in the control group.

Taste	Mean	SD	*p*-Value
Pre-test	6.35	1.73	<0.001
Post-test	9.95	2.31	

SD, standard deviation.

**Table 5:** Average test of taste sensation in the experimental group.

Taste	Mean	SD	*p*-Value
Pre-intervention	7.80	2.04	<0.001
Post-intervention	12.0	2.60	

SD, standard deviation.

**Table 6:** Comparison of percent change of olfaction and gustation between the control and experimental groups.

Measurement Tool	Percent Change		*p*-Value *
	Control Group Median (IQR)	Experimental Group Median (IQR)	
UPSIT score	14.7 (7.0–22.9)	24.1 (16.8–45.5)	0.010
TST score	60 (23.8–85.1)	52.8 (39.2–75.0)	0.820

* Mann–Whitney test; IQR, interquartile range; UPSIT, University of Pennsylvania Smell Identification Test; TST, taste strips test.

## Data Availability

The data can be provided upon reasonable request.
